# Intake of Marine-Derived Omega-3 Polyunsaturated Fatty Acids and Mortality in Renal Transplant Recipients

**DOI:** 10.3390/nu9040363

**Published:** 2017-04-05

**Authors:** António W. Gomes Neto, Camilo G. Sotomayor Campos, Ilse G. Pranger, Else van den Berg, Rijk O. B. Gans, Sabita S. Soedamah-Muthu, Gerjan J. Navis, Stephan J. L. Bakker

**Affiliations:** 1 Department of Internal Medicine, University Medical Center Groningen, University of Groningen, Hanzeplein 1, Groningen 9700 RB, The Netherlands; i.g.pranger@umcg.nl (I.G.P.); e.van.den.berg@umcg.nl (E.v.d.B.); r.o.b.gans@umcg.nl (R.O.B.G.); g.j.navis@umcg.nl (G.J.N.); s.j.l.bakker@umcg.nl (S.J.L.B.); 2 Division of Human Nutrition, Wageningen University & Research, Droevendaalsesteeg 4, Wageningen 6708 PB, The Netherlands; sabita.soedamah-muthu@wur.nl

**Keywords:** renal transplant recipients, omega-3 polyunsaturated fatty acids, cardiovascular mortality, all-cause mortality

## Abstract

The effect of marine-derived omega-3 polyunsaturated fatty acids (*n*-3 PUFA) on long-term outcome in renal transplant recipients (RTR) remains unclear. We investigated whether marine-derived *n*-3 PUFA intake is associated with all-cause and cardiovascular (CV) mortality in RTR. Intake of eicosapentaenoic acid plus docosahexaenoic acid (EPA-DHA) was assessed using a validated Food Frequency Questionnaire. Cox regression analyses were performed to evaluate the associations of EPA-DHA intake with all-cause and CV mortality. We included 627 RTR (age 53 ± 13 years). EPA-DHA intake was 102 (42–215) mg/day. During median follow-up of 5.4 years, 130 (21%) RTR died, with 52 (8.3%) due to CV causes. EPA-DHA intake was associated with lower risk of all-cause mortality (Hazard Ratio (HR) 0.85; 95% confidence interval (95% CI) 0.75–0.97). Age (*p* *=* 0.03) and smoking status (*p* = 0.01) significantly modified this association, with lower risk of all-cause and CV mortality particularly in older (HR 0.75, 95% CI 0.61–0.92; HR 0.68, 95% CI 0.48–0.95) and non-smoking RTR (HR 0.80, 95% CI 0.68–0.93; HR 0.74, 95% CI 0.56–0.98). In conclusion, marine-derived *n*-3 PUFA intake is inversely associated with risk of all-cause and CV mortality in RTR. The strongest associations were present in subgroups of patients, which adds further evidence to the plea for EPA-DHA supplementation, particularly in elderly and non-smoking RTR.

## 1. Introduction

Renal transplantation offers superior survival, quality of life, and cost-effectiveness in comparison with chronic dialysis treatment [1,2,3,4,5,6,7,8]. Thus, it is considered the “gold standard” treatment for most patients with end-stage renal disease. However, compared to age-matched controls in the general population, the survival of renal transplant recipients (RTR) continues to be significantly lower [9], with cardiovascular (CV) disease as the most important contributor to excess of mortality in RTR [10,11].

The consumption of polyunsaturated fatty acids may reduce the CV risk profile. Consequently, several countries and organizations such as the World Health Organization have made population-based diet recommendations for omega-3 polyunsaturated fatty acids (*n*-3 PUFA) intake. Fish are the major food source of the *n*-3 PUFAs docosahexaenoic acid (DHA, C22:6 *n*-3) and eicosapentaenoic acid (EPA, C20:5 *n*-3). Thus, recommendations of EPA-DHA intake can easily be met by following dietary guidelines such as those of the American Heart Association, which advises consumption of at least two fish meals per week [11,12]. 

Evidence from both epidemiologic and interventional studies have shown that EPA and DHA may exert beneficial effects on all-cause and CV mortality in both patients with pre-existing CV disease as well as in healthy populations [13,14,15,16,17,18,19]. Prospective epidemiological studies have shown that weekly fish intake is associated with lower rates of coronary heart disease (CHD) mortality in men [20,21,22]. It has also been shown in women that intake of fish and *n*-3 PUFA are inversely associated with risk of CHD death [23]. Furthermore, in a study conducted across 36 countries, fish intake was associated with a reduced risk from all-cause and ischemic heart disease mortality [24]. In addition, interventional studies have reported a reduction in all-cause mortality in male survivors of a myocardial infarction (MI), who were advised to increase their dietary intake of oily fish [25]. The largest randomized control trial testing the efficacy of *n*-3 PUFA for secondary prevention of CHD so far included 11.324 patients. It showed a 15% reduction in the primary end point of death, nonfatal MI, and nonfatal stroke, and a 20% reduction in all-cause mortality in patients receiving *n*-3 PUFA supplementation [26].

In RTR, a recent observational cohort study performed in a total of 1990 Norwegian patients found that high levels of plasma phospholipid marine *n*-3 PUFA were independently associated with better long-term survival late after renal transplantation [27]. However, this study was on plasma biomarker status and not on intake per se. Hence, a recent systematic report from The Cochrane Collaboration concluded that there is still insufficient evidence to recommend fish oil supplementation to improve survival of RTR [28]. Nonetheless, in a recent study with relatively short follow-up, we found a trend towards an inverse association of intake of marine-derived *n*-3 PUFA with all-cause mortality in RTR [29]. 

We therefore set out to update data on intake of marine-derived *n*-3 PUFA and to extend follow-up to increase power for prospective evaluation of the association of marine-derived *n*-3 PUFA intake with all-cause and CV mortality in the cohort of stable outpatient RTR. For this purpose, we investigated the intake of EPA and DHA, and evaluated its association with risk of all-cause and CV mortality in RTR. 

## 2. Methods

### 2.1. Study Design

An observational prospective study was conducted in a large single center RTR cohort [30]. All adult (≥18 years old) RTR with a functioning allograft for at least one year after transplantation and without known or apparent systemic illnesses (i.e., malignancies, opportunistic infections) who visited our outpatient clinic between November 2008 and March 2011 were invited to participate in this prospective cohort study. From a total of 817 RTR invited to be enrolled, 707 (87%) patients signed informed consent. We excluded all patients missing dietary or laboratory data, resulting in 627 RTR eligible for the statistical analysis. The present study was conducted according to the guidelines settled in the Declaration of Helsinki, and the Institutional Review Board approved the study protocol (METc 2008/186). 

The primary long-term endpoints of the current study were all-cause and CV mortality in RTR. The continuous surveillance system of the outpatient program ensures up-to-date information on patient status. We contacted general practitioners or referring nephrologists in cases the status of a patient was unknown. The patients were followed until 30 September 2015, with no loss to follow-up.

### 2.2. Renal Transplant Characteristics

RTR were all transplanted in the University Medical Center Groningen and had no history of drug or alcohol addiction according to their patient files. RTR were on standard antihypertensive and immunosuppressive therapy. Except for discouraging excess sodium intake and encouraging losing weight in overweight individuals, no specific dietary counseling was included as a routine regimen, nor was dietary recommendation regarding *n*-3 PUFA intake advised to participants. Relevant characteristics including recipient age, gender, CV history, and transplant information were extracted from patient records. Self-report questionnaires were used to obtain information on smoking behavior and alcohol intake. Physical activity was assessed using the Short Questionnaire to Assess Health-Enhancing Physical Activity (SQUASH) score in time multiplied by intensity [31].

### 2.3. Dietary Assessment

Dietary intake was assessed with a semi-quantitative validated food frequency questionnaire (FFQ) that was developed at Wageningen University [32]. The questionnaire inquired about intake of 177 food items during the last month. For each item, the frequency was documented in times per day, week, or month. The number of servings was recorded in natural units (for example, slice of bread or apple) or household measures (for example, cup or spoon). The questionnaire was self-administered and filled in at home. All FFQ were checked for completeness on the day of the visit to the outpatient clinic by a trained researcher. The results of the FFQ were converted into total energy and nutrient intake by using the Dutch Food Composition Table of 2006 [33]. 

### 2.4. Measurements

All measurements were performed once at baseline during a morning visit to the outpatient clinic. Body weight and height were measured with patients wearing indoor clothing without shoes. Body mass index (BMI) was calculated as weight in kilograms divided by height in meters squared (kg/m^2^), and body surface area (BSA) was estimated in meters squared (m^2^) [34]. Blood pressure, heart rate, and mean arterial pressure were measured according to a strict protocol and determined with a semi-automatic device (Dinamap 1846, Critikon, Tampa, FL, USA). Blood pressure and heart rate were measured every minute for 15 min, and the last three measurements were averaged [35]. 

Blood was drawn after a fasting period of 8–12 h, which included no medication intake. Diabetes was defined as use of antidiabetic medication, fasting plasma glucose ≥7.0 mmol/L [36] or glycated hemogloblin (HbA_1C_) higher than 6.5 as proposed by Shabir et al. [37]. Serum creatinine levels were measured with a modified version of the Jaffé method (MEGA AU 510, Merck Diagnostica, Darmstadt, Germany). Serum cystatin C concentrations were measured by Gentian Cystatin C Immunoassay (Gentian AS, Moss, Norway) on a Modular analyzer (Roche Diagnostics, Mannheim, Germany). Renal function was assessed by the estimated glomerular filtration rate (eGFR) based on the CKD-EPI equation of creatinine/cystatine C developed as proposed by Terpos et al. [38].

Serum albumin, high-sensitivity C-reactive protein (hs-CRP), HbA_1C_, triglycerides, low-density lipoprotein (LDL)-, high-density lipoprotein (HDL)-, and total cholesterol were measured according to routine laboratory methods. According to a strict protocol, all RTR were asked to collect a 24-h urine sample during the day before their visit to the outpatient clinic. Total urinary protein concentration was determined using the Biuret reaction (MEGA AU 150, Merck Diagnostica, Darmstadt, Germany). Proteinuria was defined as urinary protein excretion ≥0.5 g/24 h.

### 2.5. Statistical Analyses

Data analysis was performed using SPSS version 22.0 software (IBM Corp., Armonk, NY, USA), STATA version 13 (StataCorp LP, College Station, TX, USA) and R version 3.2.3 (R Foundation for Statistical Computing, Vienna, Austria). In all analyses, a two-sided *p* < 0.05 was considered significant. Continuous variables were summarized using mean (standard deviation, SD) for normally distributed data, whereas skewed distributed variables are given as median (interquartile range (IQR)); percentages were used to summarize categorical variables. Marine-derived *n*-3 PUFA intake was accounted as the sum of EPA and DHA intake (100 mg/day) adjusted for total energy intake (kJ/day) according to the residual method [39].

Linear regression analyses were performed to evaluate the association of marine-derived *n*-3 PUFA intake with baseline characteristics. Natural log transformation was used for analyses of variables with a skewed distribution. Schoenfeld residuals of EPA-DHA intake (per 100 mg) were checked: the assumption of proportional hazards was not violated (*p* = 0.65).

In prospective analyses, Cox-proportional hazard regression analyses were performed to investigate the associations of marine-derived *n*-3 PUFA intake as continuous variable, with all-cause and CV mortality in RTR. Analyses were performed with adjustment for age and sex (Model 1) and additional adjustment for eGFR, proteinuria, and time between transplantation and baseline measurement (Model 2). To avoid the inclusion of too many variables for the number of events, further models were performed with additive adjustments to model 2. We performed additional adjustment for smoking status, alcohol use, and physical activity (Model 3); for BMI, diabetes mellitus, and CV history (Model 4); for LDL-, total cholesterol, triglycerides concentration, and systolic blood pressure (Model 5); for hs-CRP and albumin concentration (Model 6). Hazard ratios (HR) of Cox-regression models are reported with 95% confidence interval (CI).

In secondary analyses, we performed stratified analyses according to subgroups of age, sex, BMI, diabetes, cardiovascular history, renal function, smoking status, alcohol use, and physical activity. We also tested for interaction to identify potential effect modification.

## 3. Results

### 3.1. Baseline Characteristics

Baseline characteristics of RTR are shown in Table 1. A total of 627 patients were included. At a median (IQR) of 5.7 (2.0–12.2) years after transplantation, participants were 53 ± 13 years old and 56.3% were male. The patients were slightly overweight (BMI 26.6 ± 4.7), and 152 (24.2%) had diabetes. Mean blood pressure of RTR was within normal boundaries (136 ± 17/83 ± 11 mmHg). It should, however, be noted that 552 (88%) of the 627 participants were using antihypertensive drugs. Mean eGFR was 45 ± 19 mL/min/1.73 m^2^ and 139 (22.2%) patients had proteinuria. Mean serum total cholesterol was 5.1 ± 1.1 mmol/L, while statins were taken by 333 (53.1%) of the RTR. Eighty-seven percent of RTR reported the use of alcohol and 12.3% were current smokers. Smoking status was unknown in 25 (4.0%) of RTR. Total energy intake was 8756 (7224–10,636) kJ/day. EPA, DHA, and DHA-EPA intake were 39 (13–85), 60 (28–129), and 102 (42–215) mg/day, respectively. 

### 3.2. Association between Intake of Marine-Derived n-3 PUFA with Clinical Baseline Characteristics

The association between EPA-DHA intake with clinical baseline characteristics is shown in Table 1. EPA-DHA intake was positively associated with age (std.β = 0.13, *p* = 0.001) and BMI (std.β = 0.08, *p* = 0.04), and was negatively associated with alcohol use (std.β = −0.14, *p* < 0.001).

Results of multivariate Cox-proportional hazard regression analyses are shown in Table 2. During follow-up of 5.4 (4.9–6.0) years, 130 (20.7%) RTR died. EPA-DHA intake was associated with lower risk of all-cause mortality (Model 2: HR 0.85; 95% CI 0.75–0.97, *p* = 0.02) independent of potential confounders including age, sex, eGFR, proteinuria, and time between transplantation and baseline. This inverse association remained significant after additional adjustment for other potential confounders. The association of EPA-DHA intake with CV mortality was of similar magnitude, but did not reach statistical significance (Model 2: HR 0.83, 95% CI 0.68–1.02, *p* = 0.08).

**Figure 1:**
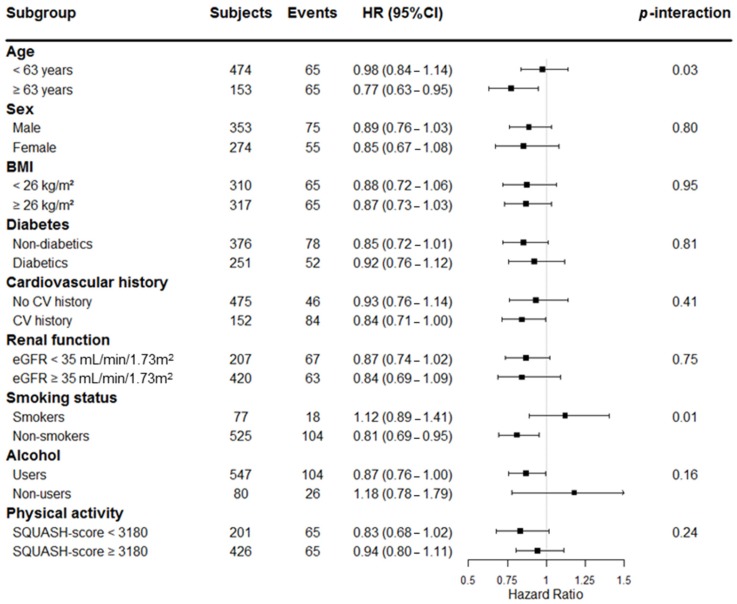
Stratified-analysis of the association of EPA-DHA intake with all-cause mortality in RTR. Hazard ratios adjusted for age and sex are shown. Subgroups with *p*-interaction < 0.05 were considered effect modifiers on the association of EPA-DHA intake with all-cause mortality.

In secondary analyses, we found effect modification of age (*p* = 0.03) and smoking status (*p* = 0.01) on the association of EPA-DHA intake with all-cause mortality, independent of age and sex (Figure 1). Consequently, we proceeded with further prospective stratified-analyses by subgroups of age (< or ≥63 years old) and smoking status (non-smokers or smokers), as depicted in Table S1 and Table S2, respectively. 

EPA-DHA intake was associated with lower risk of all-cause mortality within the older subgroup of patients (Model 2: HR 0.75; 95% CI 0.61–0.92, *p* = 0.01) but not within the younger subgroup of RTR (Model 2: HR 0.98; 95% CI 0.85–1.18, *p* = 0.8). Likewise, EPA-DHA intake was associated with lower risk of CV mortality within the older subgroup of RTR (Model 2: HR 0.68; 95% CI 0.48–0.95, *p* = 0.02), but not in the younger subgroup of patients (Model 2: HR 1.02; 95% CI 0.80–1.29, *p* = 0.9).

## 4. Discussion

In this study, we show that marine-derived *n*-3 PUFA intake is inversely associated with risk of all-cause mortality in a large cohort of stable outpatients RTR, and we show an association of similar magnitude—though not statistically significant—of marine-derived *n*-3 PUFA intake on the risk of CV mortality in RTR. We also show that age and smoking status are effect-modifiers of the association of marine-derived *n*-3 PUFA intake with all-cause mortality. Accordingly, further prospective age-stratified analysis shows that marine-derived *n*-3 PUFA intake is inversely associated with risk of all-cause and CV mortality within the older (cut-off point 63 years old), but not the younger subgroup of RTR. Likewise, prospective smoking status-stratified analysis shows that marine-derived *n*-3 PUFA intake is inversely associated with risk of all-cause and CV mortality within the non-smoker patient subgroup, but not within the subgroup of smoker RTR. 

In the general population, it has been largely shown that marine-derived *n*-3 PUFA intake may exert beneficial effects on both CV endpoints and all-cause mortality [13,14,15,16,17,18,19]. The mechanisms by which increased ingestion of *n*-3 PUFAs exert favorable effects on CV health are not fully known; however, it has been proposed that reducing susceptibility of the heart to ventricular arrhythmia, retarding growth of atherosclerotic plaques, reducing adhesion molecule expression, reducing platelet-derived growth factor, promoting nitric oxide-induced endothelial relaxation and anti-inflammatory effects may be underlying mechanisms involved [13].

Up-to-date results regarding their potential beneficial effects on survival after renal transplantation remain controversial thus far. Previously, Eide and colleagues reported that in a cohort of 1990 Norwegian RTR with 406 deaths events at a median follow-up of 6.8 years, higher plasma phospholipid marine *n*-3 PUFA levels were independently associated with better patient survival in RTR [40]. Importantly, this study was not performed on intake per se, but on plasma biomarker status of marine-derived *n*-3 PUFA, which is a resultant of intake and metabolism. However, a recent systematic report from the Cochrane Collaboration concluded that there is still insufficient evidence to recommend fish oil supplementation to improve survival of RTR [28]. On the basis of the latter statement, it seems that the beneficial effect of marine-derived *n*-3 PUFA on survival of RTR remains under discussion. Importantly, it is critical to consider that inconsistent results across the three randomized control trials accounted in this systematic review (for the outcome of mortality in RTR) may arise from methodological problems such as differing doses of fish oils, duration and timing of treatment, small number of patients, and a number of events too low to establish definitive conclusions. Previously, we reported a trend towards an inverse association of intake of marine-derived *n*-3 PUFA with all-cause mortality in RTR, with relatively short follow-up of RTR [29]. The current study updated data on *n*-3 PUFA intake and extended follow-up of our cohort. The findings of the current study support the notion [17,18,19,27] that marine-derived *n*-3 PUFAs exert beneficial effects on long-term survival after renal transplantation. Moreover, our findings support that favorable outcomes offered by marine-derived *n*-3 PUFA intake may vary among different subgroups of RTR according to age and smoking status. 

It is not completely understood which are the underlying mechanisms that explain the effect modification of age on the association of *n*-3 PUFA intake with mortality. Despite the fact that chronic low-grade inflammation remains after renal transplantation and that many of the beneficial effects of *n*-3 PUFA have been attributed to their anti-inflammatory properties [41], we did not find an association of *n*-3 PUFA intake with inflammatory biomarkers. However, it has been shown that independently of any influence on inflammation, *n*-3 PUFA may be an effective intervention against anabolic resistance [42,43,44,45], which is known to be one of the main causes of age-related sarcopenia. Animal studies have shown that *n*-3 PUFAs improve whole-body- as well as muscle insulin-sensitivity [46]. Moreover, *n*-3 PUFA supplementation helps to maintain whole-body protein metabolism via mTOR signaling pathways [47]. In healthy young adults, *n*-3 PUFA supplementation has been shown to increase the anabolic response [43], and particularly in older adults, an increase of muscle protein synthesis has been reported [42]. Thus, one might speculate that the effect modification of age on the association of *n*-3 PUFAs with mortality could be at least partly explained through the beneficial effect of *n*-3 PUFAs on muscle protein synthesis. 

Regarding smoking status as an effect modifier, it should be noted that it has previously been suggested that oral supplementation of marine-derived *n*-3 PUFA may have effects that are distinct between smokers and non-smokers [48]. Research regarding biomarkers of *n*-3 PUFA has already shown that *n*-3 PUFA biomarkers are lower in smokers. It is noteworthy that this relationship was independent of dietary intake of *n*-3 PUFAs [49]. Furthermore, it has been suggested that smoking may affect fatty acid absorption and metabolism [50], including increased *n*-3 lipid peroxidation in smokers [51,52]. Future studies are needed to better understand the effect of smoking on the metabolism of *n*-3 PUFAs. 

The strengths of our study include a median follow-up of 5.4 years with all-cause and CV mortality as clinically relevant endpoints, without loss of participants due to follow-up. Furthermore, our study included a large sample size of the specific setting of stable outpatient RTR. Moreover, data was extensively collected, which allowed adjustment for many potential confounders of the main results. On the other hand, we acknowledge that the current study has several limitations; for example, marine-derived *n*-3 PUFA intake was measured using a self-reporting FFQ, which could lead to possible over- or under-reporting of dietary intake. Moreover, dietary intake was only measured on baseline and therefore changes in dietary intake during the follow-up period might not be accounted for in our analysis. The higher the intra-individual variation of *n*-3 PUFA intake along the follow-up period would be, the greater one would expect the benefit of repeated measurement for prediction of outcomes [53,54]. Plasma biomarkers of *n*-3 PUFA were not available in this cohort study. Data regarding plasma status of marine-derived *n*-3 PUFA could have provided better understanding of the relation between dietary intake with plasma levels of marine-derived *n*-3 PUFA biomarkers. Further, CV complications or interventions were not documented; therefore, we were unable to assess the effect of dietary intake of *n*-3 PUFA on non-fatal CV-events. Additionally, our study population consisted predominantly of Caucasian people, which calls for prudence to extrapolate our results to different populations with regard to ethnicity. Finally, since the current study is observational by design, no conclusions of causality can be drawn from our results.

## 5. Conclusions

In conclusion, marine-derived *n*-3 PUFA intake is inversely associated with a lower risk of all-cause mortality in RTR. Furthermore, according to effect modifiers of this association, the beneficial effects of marine-derived *n*-3 PUFA intake are different over subgroups of RTR. Thus, marine-derived *n*-3 PUFA intake is inversely associated with all-cause and CV mortality in the older but not the younger subgroup of patients. In turn, marine-derived *n*-3 PUFA intake is associated with lower risk of all-cause and CV mortality in the non-smoker but not in the smoker subgroup of RTR. Our findings support the protective effect of marine-derived *n*-3 PUFA intake on all-cause and CV mortality that has been previously reported. We provide relevant data on intake complementary to existing data on plasma biomarkers, further adding to the evidence that they are protective. However, due to methodological heterogeneity across different studies, further design of multicenter, randomized, double-blind, placebo-controlled trials aimed at testing the beneficial effects of marine-derived *n*-3 PUFA supplementation on mortality of RTR are still lacking.

## Figures and Tables

**Table 1:** Baseline characteristics of RTR, and association of energy adjusted EPA-DHA intake across clinical parameters.

Clinical Variables	All Patients	EPA-DHA Intake (100 mg/Day)	
		Std.β	*p*
No. of patients	627	-	-
DHA, mg/day	60 (28–129)	-	-
EPA, mg/day	39 (13–85)	-	-
EPA-DHA, mg/day	102 (42–215)	-	-
Demographics			
Age, years	53 ± 13	0.13	0.001
Ethnicity (caucasian), *n* (%)	625 (99.7)	0.03	0.46
Sex (male), *n* (%)	353 (56.3)	0.04	0.39
Body mass index, kg/m^2^	26.6 ± 4.7	0.08	0.04
Body surface area, m^2^	1.9 ± 0.2	0.01	0.78
Cardiovascular history, *n* (%)	251 (40.0)	0.04	0.28
Renal transplantation characteristics			
Pre-emptive transplantation, *n* (%)	102 (16.3)	0.003	0.93
Time between transplantation and baseline, years	5.7 (2.0–12.2)	−0.002	0.95
Hemodynamic parameters			
Systolic blood pressure, mmHg	136 ± 17	0.001	0.98
Diastolic blood pressure, mmHg	83 ± 11	0.03	0.41
Mean arterial pressure, mmHg	108 ± 15	0.02	0.71
Heart rate, beats per minute	69 ± 12	0.01	0.78
Antihypertensives, *n* (%)	552 (88.0)	−0.04	0.34
Renal function parameters			
Creatinine, umol/L	123 (99–159)	0.005	0.91
Cystatine-C, mg/L	1.7 (1.3–2.2)	−0.06	0.13
eGFR, mL/min/1.73 m^2^	45 ± 19	−0.01	0.83
Proteinuria ≥0.5 g/day, *n* (%)	139 (22.2)	−0.02	0.55
Glucose homeostasis			
Glucose, mmol/L	5.3 (4.8–6.0)	0.06	0.14
HbA_1C_, %	5.8 (5.5–6.2)	0.05	0.27
Diabetes, *n* (%)	152 (24.2)	0.02	0.60
Antidiabetic medication, *n* (%)	98 (15.6)	0.02	0.67
Serum parameters			
Albumin, g/L	43.0 ± 3.0	−0.02	0.67
hs-CRP, mg/L	1.6 (0.7–4.5)	0.06	0.13
Lipids			
Total cholesterol, mmol/L	5.1 ± 1.1	0.08	0.06
LDL cholesterol, mmol/L	3.0 ± 0.9	0.06	0.13
HDL cholesterol, mmol/L	1.3 (1.1–1.7)	0.07	0.10
Triglycerides, mmol/L	1.7 (1.2–2.3)	−0.01	0.75
Statin use, *n* (%)	333 (53.1)	0.02	0.57
Health lifestyle			
Current smoker, *n* (%)	77 (12.3)	−0.008	0.85
Alcohol consumers, *n* (%)	547 (87.2)	−0.14	<0.001
Physical activity, intensity × hours	5250 (2400–8160)	0.03	0.41
Total energy intake, kJ/day	8756 (7224–10,636)	-	-

RTR, renal transplant recipients; EPA, eicosapentaenoic acid; DHA, docosahexaenoic acid; eGFR, estimated glomerular filtration rate; HbA_1C_, glycated hemoglobin; hs-CRP, high-sensitivity C-reactive protein; LDL, low-density lipoprotein; HDL, high-density lipoprotein; kJ, kilojoule.

**Table 2:** Prospective analysis of EPA-DHA intake (100 mg/day) on all-cause and CV mortality in RTR.

Model	EPA-DHA Intake, 100 mg/Day	
	HR (95% CI)	*p*
All-cause mortality		
Model 1	0.87 (0.77–0.99)	0.03
Model 2	0.85 (0.75–0.97)	0.02
Model 3	0.87 (0.77–1.00)	0.04
Model 4	0.87 (0.76–0.99)	0.03
Model 5	0.84 (0.73–0.96)	0.01
Model 6	0.85 (0.74–0.97)	0.02
CV mortality		
Model 1	0.85 (0.69–1.05)	0.13
Model 2	0.83 (0.68–1.02)	0.08
Model 3	0.86 (0.70–1.07)	0.18
Model 4	0.84 (0.68–1.03)	0.10
Model 5	0.81 (0.64–1.01)	0.06
Model 6	0.82 (0.65–1.03)	0.08

RTR, renal transplant recipients; EPA, eicosapentaenoic acid; DHA, docosahexaenoic acid; CV, cardiovascular. Model 1: adjustment for age and sex. Model 2: model 1 + adjustment for estimated glomerular filtration Rate, proteinuria, and time between transplantation and baseline measurement. Model 3: model 2 + adjustment for smoking status, alcohol use, and physical activity. Model 4: model 2 + adjustment for body mass index, diabetes mellitus and cardiovascular history. Model 5: model 2 + adjustment for total cholesterol, low-density lipoprotein-cholesterol, triglycerides concentration, and systolic blood pressure. Model 6: model 2 + adjustment for high-sensitivity C-reactive protein and albumin concentration.

## Supplementary Materials

The following are available online at http://www.mdpi.com/2072-6643/9/4/363/s1, Table S1: Prospective age-stratified analysis of EPA-DHA intake (100 mg/day) on all-cause and CV mortality in RTR, Table S2: Prospective smoking status-stratified analysis of EPA-DHA intake (100 mg/day) on all-cause and CV mortality in RTR.

## Abbreviations

BSA	Body surface area
CI	Confidence interval
CV	Cardiovascular
DHA	Docosahexaenoic acid
EPA	Eicosapentaenoic acid
EPA-DHA	Eicosapentaenoic acid plus Docosahexaenoic acid
FFQ	Food frequency questionnaire
HDL	High-density lipoprotein
HR	Hazard Ratio
HbA_1C_	Glycated hemoglobin
hs-CRP	High-sensitive C-reactive protein
IQR	Interquartile range
kJ	Kilojoule
LDL	Low-density lipoprotein
*n*-3 PUFA	Omega-3 polyunsaturated fatty acids
RTR	Renal transplant recipients
SQUASH	Short QUestionnaire to ASsess Health-enhancing physical activity
