# Erratum: Intake of Marine-Derived Omega-3 Polyunsaturated Fatty Acids and Mortality in Renal Transplant Recipients; *Nutrients* 2017, *9*, 363

**DOI:** 10.3390/nu9060614

**Published:** 2017-06-16

**Authors:** Nutrients Editorial Office

**Affiliations:** MDPI AG, St. Alban-Anlage 66, 4052 Basel, Switzerland; nutrients@mdpi.com

Due to a mistake during the production process, there was a spelling error in one of the author names in the original published version [1]. The *Nutrients* Editorial Office therefore wishes to make this correction to the paper:

The name of the second author “Camilo G. Sotomayor Campos” was corrected to “Camilo G. Sotomayor”.

We would like to apologize for any inconvenience caused to the authors and readers by this mistake. We will update the article and the original version will remain available on the article webpage.
