# Supplementary material for: Intake of Marine-Derived Omega-3 Polyunsaturated Fatty Acids and Mortality in Renal Transplant Recipients
Source: Nutrients. 2017 Apr 5;9(4):363. doi: 10.3390/nu9040363 (PMC5409702; doi:10.3390/nu9040363)
Supplement: Supplementary file 1 [file nutrients-09-00363-s001.docx]

**Table S1.** Prospective age-stratified analysis of EPA-DHA intake (100 mg/day) on all-cause and CV mortality in RTR.

| **Type** | **EPA-DHA Intake, 100 mg/day** | | | |
| --- | --- | --- | --- | --- |
|  | <63 years old | | ≥63 years old | |
| No. of subjects | 474 | | 153 | |
|  | HR (95 % CI) | *p* | HR (95 % CI) | *p* |
| No. of events | 65 |  | 65 |  |
| Model 1 | 0.98 (0.84–1.14) | 0.8 | 0.77 (0.63–0.95) | 0.01 |
| Model 2 | 0.98 (0.84–1.15) | 0.8 | 0.75 (0.61–0.91) | 0.01 |
| Model 3 | 0.98 (0.86–1.17) | 0.9 | 0.78 (0.63–0.96) | 0.02 |
| Model 4 | 0.98 (0.84–1.15) | 0.8 | 0.75 (0.61–0.93) | 0.01 |
| Model 5 | 0.97 (0.82–1.14) | 0.7 | 0.73 (0.59–0.90) | 0.004 |
| Model 6 | 0.98 (0.83–1.17) | 0.8 | 0.73 (0.58–0.91) | 0.004 |
| No. of events | 25 |  | 27 |  |
| Model 1 | 1.01 (0.80–1.27) | 0.9 | 0.70 (0.50–1.00) | 0.05 |
| Model 2 | 1.02 (0.80–1.29) | 0.9 | 0.68 (0.48–0.95) | 0.02 |
| Model 3 | 1.04 (0.82–1.32) | 0.8 | 0.68 (0.47–0.99) | 0.05 |
| Model 4 | 1.01 (0.80–1.26) | 0.9 | 0.66 (0.47–0.95) | 0.02 |
| Model 5 | 0.97 (0.74–1.26) | 0.8 | 0.67 (0.46–0.98) | 0.04 |
| Model 6 | 1.02 (0.78–1.31) | 0.9 | 0.65 (0.44–0.96) | 0.03 |

RTR, renal transplant recipients; EPA, Eicosapentaenoic acid; DHA, Docosahexaenoic acid; CV, cardiovascular. Model 1: adjustment for age and sex. Model 2: model 1 + adjustment for estimated Glomerular Filtration Rate, proteinuria and time between transplantation and baseline measurement. Model 3: model 2 + adjustment for smoking status, alcohol use, and physical activity. Model 4: model 2 + adjustment for body mass index, diabetes mellitus and cardiovascular history. Model 5: model 2 + adjustment for total cholesterol, low-density lipoprotein-cholesterol, triglycerides concentration, and systolic blood pressure. Model 6: model 2 + adjustment for high-sensitivity C-reactive protein and albumin concentration.

**Table S2.** Prospective smoking status-stratified analysis of EPA-DHA intake (100 mg/day) on all-cause and CV mortality in RTR.

| **Type** | **EPA-DHA Intake, 100 mg/day** | | | |
| --- | --- | --- | --- | --- |
|  | Non-smokers | | Smokers | |
| No. of subjects | 525 | | 77 | |
|  | HR (95 % CI) | *p* | HR (95 % CI) | *p* |
| All-cause mortality | | | | |
| No. of events | 104 |  | 18 |  |
| Model 1 | 0.81 (0.69–0.95) | 0.01 | 1.12 (0.89–1.41) | 0.3 |
| Model 2 | 0.80 (0.68–0.93) | 0.01 | 1.15 (0.89–1.48) | 0.3 |
| Model 3 | 0.81 (0.70–0.96) | 0.01 | 1.15 (0.88–1.50) | 0.3 |
| Model 4 | 0.80 (0.68–0.94) | 0.01 | 1.18 (0.88–1.57) | 0.3 |
| Model 5 | 0.80 (0.68–0.94) | 0.01 | 1.11 (0.82–1.50) | 0.5 |
| Model 6 | 0.78 (0.66–0.92) | 0.003 | 1.23 (0.91–1.66) | 0.2 |
| CV mortality | | | | |
| No. of events | 38 |  | 9 |  |
| Model 1 | 0.76 (0.57–1.01) | 0.05 | 1.15 (0.85–1.57) | 0.4 |
| Model 2 | 0.74 (0.56–0.98) | 0.04 | 1.16 (0.80–1.69) | 0.4 |
| Model 3 | 0.76 (0.57–1.00) | 0.05 | 1.24 (0.83–1.84) | 0.3 |
| Model 4 | 0.73 (0.55–0.97) | 0.03 | 1.17 (0.79–1.73) | 0.4 |
| Model 5 | 0.76 (0.57–1.02) | 0.06 | 1.03 (0.64–1.67) | 0.9 |
| Model 6 | 0.68 (0.50–0.94) | 0.02 | 1.21 (0.76–1.92) | 0.4 |

RTR, renal transplant recipients; EPA, Eicosapentaenoic acid; DHA, Docosahexaenoic acid; CV, cardiovascular. Model 1: adjustment for age and sex. Model 2: model 1 + adjustment for estimated Glomerular Filtration Rate, proteinuria and time between transplantation and baseline measurement. Model 3: model 2 + adjustment for alcohol use, and physical activity. Model 4: model 2 + adjustment for body mass index, diabetes mellitus and cardiovascular history. Model 5: model 2 + adjustment for total cholesterol, low-density lipoprotein-cholesterol, triglycerides concentration, and systolic blood pressure. Model 6: model 2 + adjustment for high-sensitivity C-reactive protein and albumin concentration.
